# Embracing discordance: Phylogenomic analyses provide evidence for allopolyploidy leading to cryptic diversity in a Mediterranean *Campanula* (Campanulaceae) clade

**DOI:** 10.1111/evo.13203

**Published:** 2017-02-25

**Authors:** Andrew A. Crowl, Cody Myers, Nico Cellinese

**Affiliations:** ^1^ Florida Museum of Natural HistoryUniversity of FloridaGainesvilleFlorida32611; ^2^ Department of BiologyUniversity of FloridaGainesvilleFlorida32611

**Keywords:** Campanulaceae, cryptic diversity, hybridization, Mediterranean Basin, phylogenomics, polyploidy

## Abstract

The Mediterranean Basin harbors a remarkable amount of biodiversity, a high proportion of which is endemic to this region. Here, we present an in‐depth study of an angiosperm species complex, in which cryptic taxonomic diversity has been hypothesized. Specifically, we focus on four currently recognized species in the *Roucela* complex, a well‐supported clade in the Campanulaceae/Campanuloideae: *Campanula creutzburgii*, *C. drabifolia*, *C. erinus*, and *C. simulans*. This study takes a phylogenomic approach, utilizing near‐complete plastomes and 130 nuclear loci, to uncover cryptic diversity and test hypotheses regarding hybridization and polyploidy within this clade. Genome size estimates recovered tetraploid and octoploid lineages within the currently recognized, widespread species *C. erinus*, showing an east‐west geographic pattern. Though genomic data clearly differentiate these two cytotypes, we failed to discern morphological differences. The formation of a cryptic octoploid lineage, distributed across the eastern Mediterranean, is hypothesized to be the result of an allopolyploid event in which one parental morphology is retained. The tetraploid *C. erinus* and *C. creutzburgii* (also a tetraploid) are implicated as parental lineages. Our results highlight the utility of target‐enrichment approaches for obtaining genomic datasets for thorough assessments of species diversity and the importance of carefully considering gene‐tree discordance within such datasets.

Cryptic species are those for which a lack of morphological differentiation has hindered recognition of genetically distinct lineages. Accurate species delimitation is a difficult but critical aspect of systematics because, although the subject of much debate, species are regarded as fundamental units of biogeography, ecology, and conservation. As threats to biodiversity mount, accurate assessments are increasingly important as inaccurate delimitation of species may hinder biological inferences and conservation efforts (Wiens 2007). In this study, we focus on a group of endemic Mediterranean plants, in which cryptic diversity has been postulated (Crowl et al. 2015).

The Mediterranean Basin is among the most biologically diverse areas in the world, harboring innumerable poorly understood, species‐rich groups (Myers 1990; Médail and Quézel 1997). Understanding evolutionary processes and species diversity is of special interest in this region given the exceptionally high degree of endemism and proportion of rare and threatened taxa (Greuter 1991; Médail and Quézel 1997).

The *Roucela* clade (Campanulaceae: Campanuloideae) comprises 12 currently recognized, mostly narrowly endemic species of bellflowers found primarily in the eastern Mediterranean Basin (Carlström 1986; Crowl et al. 2015). These taxa exhibit a high degree of endemism within this region, often confined to a single or a few islands, with the exception of *Campanula erinus*. As currently recognized, this taxon is distributed across the Mediterranean Basin from the Arabian Peninsula to Macaronesia. Baker's Law, which states that self‐compatible individuals are more likely to be successful colonizers following a long‐distance dispersal event than self‐incompatible individuals (Baker 1955), may provide insights into this pattern. This taxon's ability to self‐pollinate (Carlström 1986) likely explains the abnormally broad distribution pattern of *C. erinus* within an otherwise highly endemic (and self‐incompatible) plant clade.

Recent phylogenetic analyses (Crowl et al. 2015) recovered strong support for evolutionary relationships of these taxa, with the exception of one clade. These analyses, based on five plastid and two nuclear loci, failed to disentangle the group containing the Cretan endemic, *C. creutzburgii*, *C. drabifolia*, endemic to the mainland of Greece, *C. simulans* from southwestern Turkey, and the most widespread taxon, *C. erinus*. Of these, only *C. simulans* was strongly supported as monophyletic. To increase phylogenetic resolution within this clade and test hypotheses regarding hybridization and cryptic diversity, we increased population sampling of each taxon and constructed a genomic dataset comprising 130 nuclear loci and near‐complete plastomes for 105 individuals.

We used these datasets to test hypotheses concerning the nature of previously unrecognized diversity within the *Roucela* clade in the Mediterranean Basin, as suggested by a previous study (Crowl et al. 2015). Specifically, results from numerous phylogenetic analyses, including concatenation and species‐tree approaches, suggest two cryptic lineages within the currently recognized, widespread species, *C. erinus*. These lineages are consistent with both geography and ploidy, with a tetraploid clade consisting of populations found in the western Mediterranean Basin and an octoploid lineage restricted to the eastern portion of the basin (Fig. 1). Network analyses, corroborated by nuclear gene‐tree topologies, indicate a hybrid origin for the octoploid. The narrow Cretan endemic, *C. creutzburgii* (a tetraploid) and the western Mediterranean *C. erinus* (also a tetraploid) are implicated as parental lineages.

**Figure 1:**
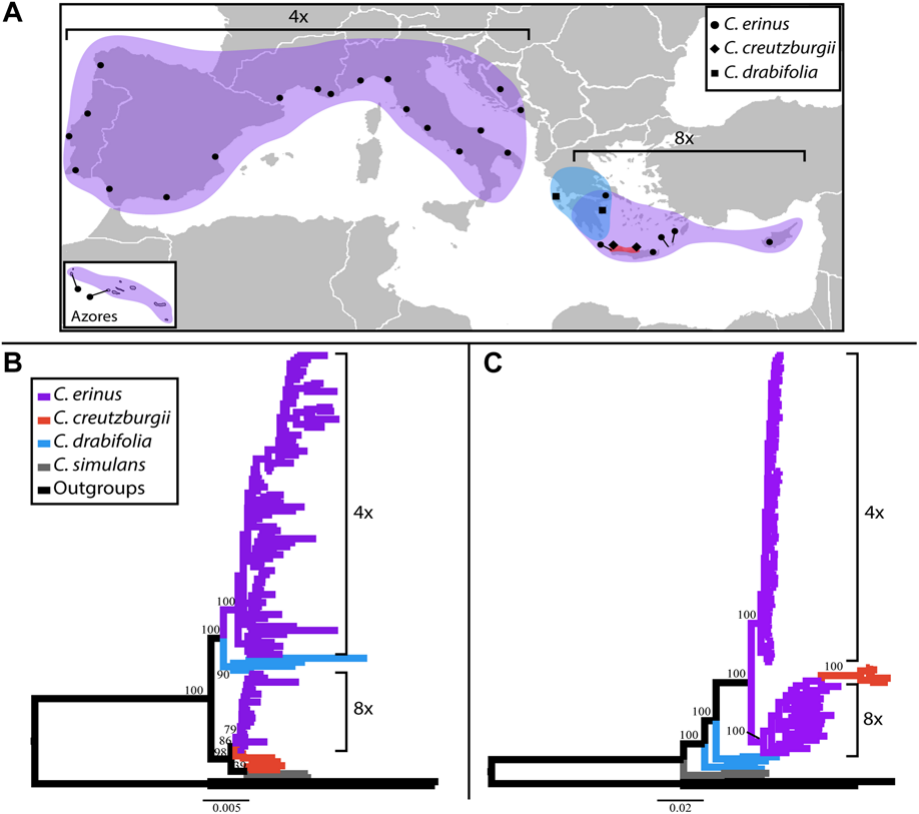
Sample localities and comparison of concatenated analyses. (A) Occurrence map of lineages under investigation. Colors are consistent with those in panels B and C, with tetraploid and octoploid *Campanula erinus* lineages labeled as such on the map. Circles indicate sampled populations of *C. erinus*, diamonds indicate sampling localities of *C. creutzburgii* populations, and squares indicate sampling localities of *C. drabifolia* populations included in this study. (B) Results from maximum likelihood analysis of plastome dataset. Numbers at nodes indicate bootstrap support for relationships and clades discussed in the text. Scale bar indicates nucleotide substitutions per site. (C) Results from maximum likelihood analysis of concatenated nuclear dataset. Numbers at nodes indicate bootstrap support for relationships and clades discussed in the text.

The target enrichment approach taken in this study resulted in a powerful genomic dataset used to uncover previously overlooked diversity in the eastern Mediterranean Basin. Accurate species assessments in this hotspot of biodiversity are especially critical in the face of future climate change, which is projected to increase aridity in the Mediterranean Basin (e.g., Gao and Giorgi 2008) and, thus, further impact subtropically adapted taxa such as the *Roucela* clade (Crowl et al. 2015).

## Materials and Methods

### SAMPLING

Taxon sampling included 105 representatives from six species in the *Roucela* clade. We sampled 2–5 individuals from 27 populations spanning the distribution of *Campanula erinus* from the Azores to Cyprus (Fig. 1; accession data provided in Supporting Information). Four individuals of *C. creutzburgii*, four individuals of *C. drabifolia*, and two individuals of *C. simulans* were also included. On the basis of recent phylogenetic results (Crowl et al. 2015), two additional species in the *Roucela* clade were used as outgroups: *C. rhodensis* and *C. lycica*. DNA was extracted from silica‐dried and herbarium material following a modified CTAB extraction protocol (Doyle and Doyle 1987). Data, including alignments and gene trees, available from the Dryad Digital Repository: https://doi.org/10.5061/dryad.hn06n

### MOLECULAR DATA

Previous analyses using plastid data and a small number of nuclear loci indicated difficulty in resolving phylogenetic relationships of *Campanula erinus*, *C. creutzburgii*, *C. drabifolia*, and *C. simulans* (Mansion et al. 2014; Crowl et al. 2014; Crowl et al. 2015). We, therefore, obtained a large, multilocus nuclear dataset and plastome dataset to disentangle species relationships. This was achieved using a sequence capture approach (Cronn et al. 2012; Mandel et al. 2014) for the purpose of target enrichment prior to sequencing the reduced‐representation libraries.

MarkerMiner (Chamala et al. 2015) was used to discover and develop probes for single‐copy nuclear loci. We used four assembled Campanulaceae transcriptomes (*Lobelia siphilitica*, *Platycodon grandiflorus*, *Campanula delicatula*, and *Campanula erinus*; available through the 1KP project; onekp.com). This method uses reciprocal BLAST (Altschul et al. 1997) searches, a database of single‐copy nuclear genes (De Smet et al. 2013), and clustering steps to identify putative orthologous and single‐copy loci across input sequences. *Arabidopsis thaliana* was used as a reference to estimate intron/exon boundaries.

Probes were designed for 246 nuclear loci, ranging from 120–3680 bp in length, conserved across the *Roucela* clade. In‐solution biotinylated probes were synthesized using a custom MYbaits target enrichment kit (MYcroarray, Ann Arbor, MI; http://www.microarray.com). The 120mer probes (10,000 total baits) were used with 2× tiling density. Additionally, we isolated 90 putatively single‐copy nuclear genes conserved across all input transcriptomes, useful for future studies in the Campanulaceae. Library building and capture reactions were carried out by RAPiD Genomics (Gainesville, FL; http://www.rapid-genomics.com). Dual indexed libraries were prepared with adapters containing i7‐i5 indices. Samples were sequenced using the Illumina HiSeq 3000 platform (2 × 100 reads).

### DATA PROCESSING

Quality filtering of Illumina reads was carried out using cutadapt (Martin 2011) and sickle (Joshi 2011) to remove adapter sequences and trim low‐quality nucleotides. Default parameters were used. The HybPiper pipeline (v.1.0; Johnson et al. 2016) was then used to assemble loci. This pipeline uses BWA (Li and Durbin 2009) to align reads to target sequences and SPAdes (Bankevich et al. 2012) to assemble these reads into contigs. If multiple contigs that contained sequences representing at least 75% of the original bait length were found, these were flagged as potential paralogs and all copies were removed from downstream analyses. A further filtering step was conducted by manual inspection of gene trees (see *Phylogenetic analysis* below) to remove paralogous loci that may have been missed by the first filtering step.

Consensus contigs were aligned to the original probe sequences. The resulting loci were not trimmed to the original probe length, however. This allowed the sequences to extend into putative intronic regions. After quality filtering and removal of potential paralogous loci, 130 loci contained orthologous sequences for all 109 sampled taxa (no missing data).

Plastomes were assembled in a similar way, using *Trachelium caeruleum* (Haberle et al. 2008) as a reference. Aligned contigs were trimmed to the plastome reference length.

### PHYLOGENETIC ANALYSIS

Individual gene and plastome alignments were constructed using MAFFT (v.7.245; Katoh et al. 2002, 2013). Plastomes were considered as a single locus for all subsequent analyses. We estimated individual nuclear gene trees as well as a concatenated phylogeny using maximum likelihood (ML) with the program RA × ML (v.7.3.2; Stamatakis 2006). The ML searches were run using 10 distinct starting trees and 1000 bootstrap replicates to measure support. PartitionFinder (v.2.0.0; Lanfear et al. 2012) was used to infer the optimal partitioning schemes and models of molecular evolution for the alignments using the *rcluster* search option.

Initial results indicated a number of samples with inconsistent and contradictory phylogenetic placement (referred to as rogue taxa) present in the nuclear dataset. We used Rogue NaRok (Aberer et al. 2013) to identify such OTUs. This analysis, optimized for support using a majority rule consensus threshold, identified four individuals of *C. erinus* as rogue samples. These accessions were removed from all datasets and the ML analyses were rerun as above.

### COALESCENT SPECIES‐TREE ANALYSES

The relatively young age of the *C. erinus* complex suggests lineage sorting has the potential to confound results from concatenation approaches (Crowl et al. 2015). We, therefore, utilized recently developed coalescent methods to estimate a species tree for the clade in this study.

ASTRAL‐II (v.4.10.0; Mirarab and Warnow 2015), which estimates the species tree that maximizes the number of shared quartet trees given a set of gene trees, has been found to be consistent and accurate in simulations compared to alternative coalescent approaches (Mirarab et al. 2014). The 130 ML gene trees (best trees) inferred using RA × ML were used as input, and local posterior probabilities were estimated to provide support for relationships. With respect to individuals being assigned to “species” in the allele table, two approaches were taken: (1) A population tree was estimated by assigning individuals to separate populations; (2) *C. erinus* individuals were assigned to eastern‐Mediterranean (octoploid) and western‐Mediterranean (tetraploid) lineages while *C. drabifolia* and *C. creutzburgii* populations were kept separate, as suggested by the ML analyses.

Additionally, we used SVDquartets (Chifman and Kubatko 2014) implemented in PAUP* (v.4.0a147; Swofford 2002) to verify results generated by ASTRAL‐II. A coalescent approach originally intended for SNP data, SVDquartets has been shown to perform well on multilocus datasets despite violating the assumption that sites are independent (Chifman and Kubatko 2014). We used the concatenated nuclear data matrix as input, evaluated 100,000 random quartets, and assessed support using 100 bootstrap replicates.

### BAYESIAN CONCORDANCE ANALYSIS

Though current species‐tree methods assume no migration between populations (Heled and Drummond 2010; Bryant et al. 2012), concordance analyses can still recover primary phylogenetic signal in the presence of gene flow (Larget et al. 2010). We, therefore, summarized topological concordance among loci using BUCKy (v.1.4.4; Baum 2007; Ane et al. 2007; Larget et al. 2010). Due to computational constraints, it was necessary to reduce our molecular dataset to the eight major lineages recovered in previous analyses. We chose individuals at random to represent the eight lineages. A second iteration of this, carried out using a different random sampling of individuals, verified the results were not affected by which representative samples were present in the dataset. Individual gene trees for this analysis were estimated using MrBayes (v.3.2; Ronquist et al. 2012). To test the impact of the discordance parameter (alpha), independent analyses were run using alpha = 1, alpha = 10, and alpha = 1000. All analyses were run with four Markov chain Monte Carlo (MCMC) chains for 1 million generations. Burn‐in was set to 10%.

### GENE‐TREE BINNING

To better understand the nature of discordance within our dataset, we carried out a manual inspection of individual gene trees. Nuclear gene trees were binned according to whether they fell into one of two topologies being recovered: (1) monophyletic tetraploid and octoploid *C. erinus* lineages (consistent with the ASTRAL‐II and SVDquartets topologies); or (2) the octoploid *C. erinus* lineage sister to *C. creutzburgii* (consistent with the BUCKy and plastome topologies).

### NETWORK ANALYSIS

We further explored the possibility of hybridization using the program SNaQ (Solís‐Lemus and Ané 2016). This approach estimates a phylogenetic network under the coalescent model to account for incomplete lineage sorting, while allowing for reticulation events within a pseudo‐likelihood framework. In order to infer the species network, we obtained a table of quartet concordance factors from our previous BUCKy analysis using the *bucky.pl* script provided as part of the TICR pipeline (https://github.com/nstenz/TICR). This is achieved by summarizing the output files from MrBayes for each locus, calculating all possible 4‐taxon sets (quartets), and running each through BUCKy. Tetraploid and octoploid *C. erinus* populations were regarded as separate lineages as in all previous species‐tree estimations. We ran two separate analyses, allowing one (hmax = 1) and two (hmax = 2) hybridization events. Both were executed with 10 independent runs on a starting tree (reduced topology from nuclear concatenated analysis).

### PLOIDY ESTIMATION

Chromosome counts were obtained from the literature (Carlström 1986). To infer ploidy of the 27 *C. erinus* populations included in our phylogenetic analyses, we used a modified flow cytometry method described in Roberts et al. (2009). Approximately 4 mg of recently collected, silica‐dried sample material was combined with 2 mg of *Pisum sativum* as an internal standard in a 1.5‐mL Eppendorf tube containing 2–3 zirconia beads and placed in a bead mill for 1–3 seconds. Cold lysis buffer (500 μl) was added to the ground material and filtered through cell culture tubes. RNaseA (1 μl) was then added. Finally, 35 μl of Propidium Iodide staining solution was added to each tube of suspended nuclei. Samples were run on a BD Accuri C6 flow cytometer (BD Biosciences, San Jose, California) at the University of Florida. Two to five individuals per population were used to confirm ploidy estimates.

### MORPHOLOGY

To determine if there were measurable morphological differences between the two lineages of *C. erinus* identified by molecular data, we focused on bract teeth, a morphological feature found to be taxonomically informative within the *Roucela* complex (see Fig. 4 panel B for illustration). Carlström (1986) showed that the length of the bract teeth unambiguously distinguishes *C. creutzburgii* from *C. erinus*. This feature has the added advantage of being well preserved in herbarium specimens, regardless of specimen age or preservation method, as opposed to floral characteristics, which do not preserve well in this group. Using ImageJ (v.2.0.0), we measured bract length, bract area, and bract tooth length for 22 digitized specimens of *C. creutzburgii*, 123 tetraploid *C. erinus* individuals, and 129 octoploid *C. erinus* individuals across their ranges. To account for tooth length differences due to confounding factors such as age of the plant or bract age on individual plants, we corrected tooth measurements by dividing these values by the length of the entire bract.

## Results

### PHYLOGENETIC ANALYSES

Though plastid regions were not targeted in our probe design, we obtained a significant amount of data from the plastid genome as a byproduct of the Illumina sequencing run. We recovered between 83.1% and 99.7% (avg. = 95.7%) plastome coverage for all samples. Maximum likelihood analysis of the plastome dataset found strong support for two *Campanula erinus* clades (Fig. 1). Tetraploid populations were maximally supported as monophyletic and sister to *C. drabifolia*. Octoploid populations were moderately supported (BS = 86) as monophyletic and sister to two individuals of *C. creutzburgii* from western Crete. The placement of the remaining *C. creutzburgii* samples was not supported but inferred to be sister to a monophyletic *C. simulans*.

ML analysis of the concatenated nuclear dataset recovered maximal support for all relationships discussed below. A monophyletic *C. creutzburgii* was nested within the octoploid *C. erinus* populations, rendering the octoploids paraphyletic. A tetraploid *C. erinus* clade was inferred to be sister to this octoploid plus *C. creutzburgii* assemblage. *Campanula drabifolia* was found to be nonmonophyletic. *Campanula simulans* was again recovered as monophyletic and sister to the rest.

Individual nuclear gene trees showed high levels of phylogenetic discordance. Close inspection of gene trees indicated two major topologies were being recovered. Approximately 30% of nuclear loci indicated tetraploid and octoploid populations of *C. erinus* were reciprocally monophyletic (consistent with the ASTRAL‐II and SVDquartets species‐tree analyses; see below). The second topology, recovered with approximately 34% of genes, indicated the octoploid lineage was sister to *C. creutzburgii*. This was consistent with the plastome dataset and BUCKy analyses (see below). The remainder (36%) of the gene trees did not fall into these strictly defined categories. However, when we accounted for low statistical support (BS < 80) for relationships of the *C. erinus* lineages in these gene trees, we found that nearly all of them approximated one of the two previously discussed topologies. This more relaxed assignment of gene trees suggested that nearly half of the sampled genome approximated the plastome‐like topology (close association of the octoploid lineage with *C. creutzburgii*; 43%), while the other half (49%) indicated a close association between the octoploid and tetraploid *C. erinus* populations.

### SPECIES‐TREE AND NETWORK ANALYSES

**Figure 2:**
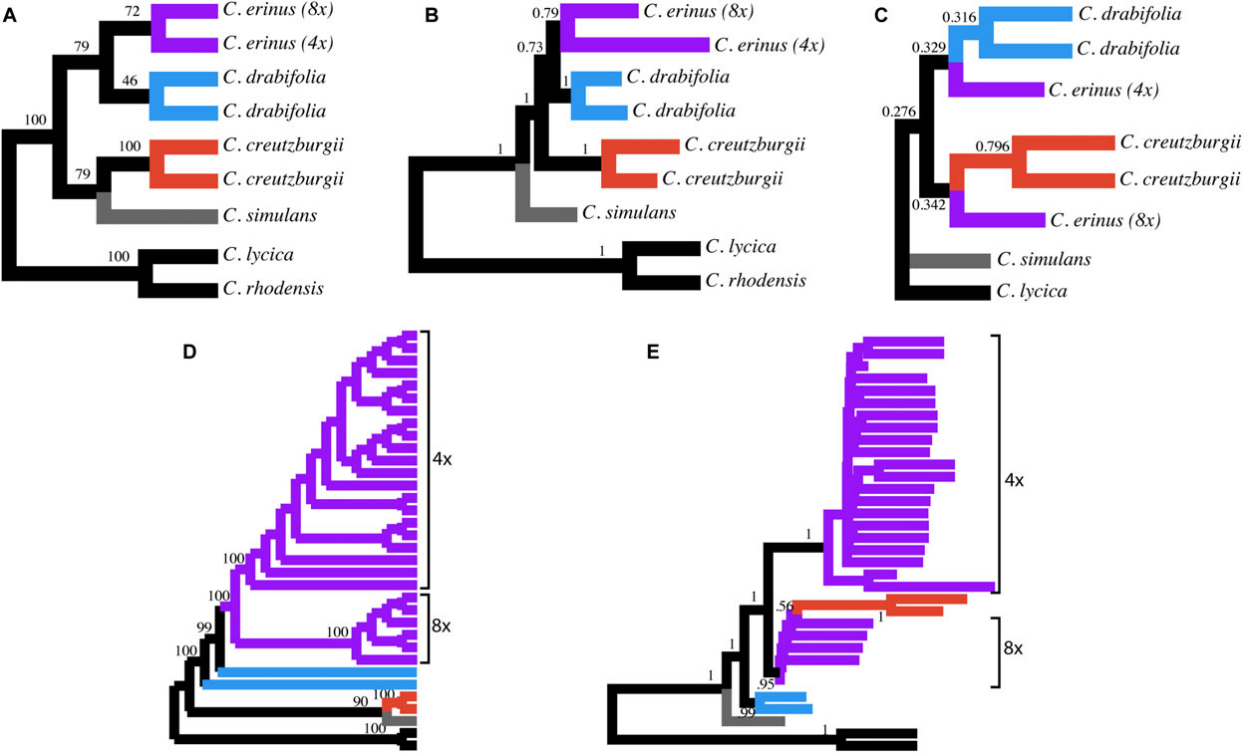
Comparison of species‐tree analyses. (A) Species‐tree topology estimated with SVDquartets when individuals were assigned to the major lineages recovered with concatenation analyses (see Fig. 1). Numbers at nodes indicate bootstrap support values of relationships. (B) Species‐tree topology estimated with ASTRAL‐II when individuals were assigned to lineages, as in panel A. (C) Primary concordance tree from Bayesian Concordance Analysis in Bucky. Numbers indicate concordance factors and are, thus, estimates of the proportion of the genome for which a relationship is true. (D) Topology estimated with SVDquartets when individuals were assigned to separate populations, rather than the major lineages as in panel A. Numbers indicate bootstrap support values. (E) Topology estimated with ASTRAL‐II when individuals were assigned to separate populations. Numbers indicate local posterior probability support values. Branch lengths shown in coalescent units.

Species‐tree analyses of the nuclear dataset recovered relationships consistent with the individual gene trees but differed from the ML concatenation results. Both ASTRAL‐II and SVDquartets recovered species trees in which tetraploid and octoploid *C. erinus* lineages were reciprocally monophyletic when individuals were assigned to lineages (Fig. 2, panels A and B). Support for the *C. erinus* sister relationship, however, was low (PP = 0.79 in ASTRAL‐II; BS = 72 in SVDquartets). Bayesian concordance analysis, as implemented in BUCKy, inferred a primary concordance tree in which the octoploid *C. erinus* lineage was sister to *C. creutzburgii*, while the tetraploid *C. erinus* lineage was sister to *C. drabifolia* (Fig. 2, panel C), consistent with the plastome phylogeny. These relationships had concordance factors of CF = 0.342 and CF = 0.329, respectively (see also results from manual inspection of gene trees above).

Interestingly, ASTRAL‐II and SVDquartets differed in the reconstruction of the specie‐tree topology when individuals were assigned to populations, rather than assigning them to the lineages discussed above. When we used the population assignments, ASTRAL‐II estimated a topology similar to the concatenation analyses, with *C. creutzburgii* populations nested within octoploid *C. erinus* populations (Fig. 2, panel E). There was, however, very low support for this relationship. The SVDquartets analysis using population assignments strongly supported a monophyletic octoploid *C. erinus*, and *C. creutzburgii* sister to *C. simulans* (Fig. 2, panel D).

**Figure 3:**
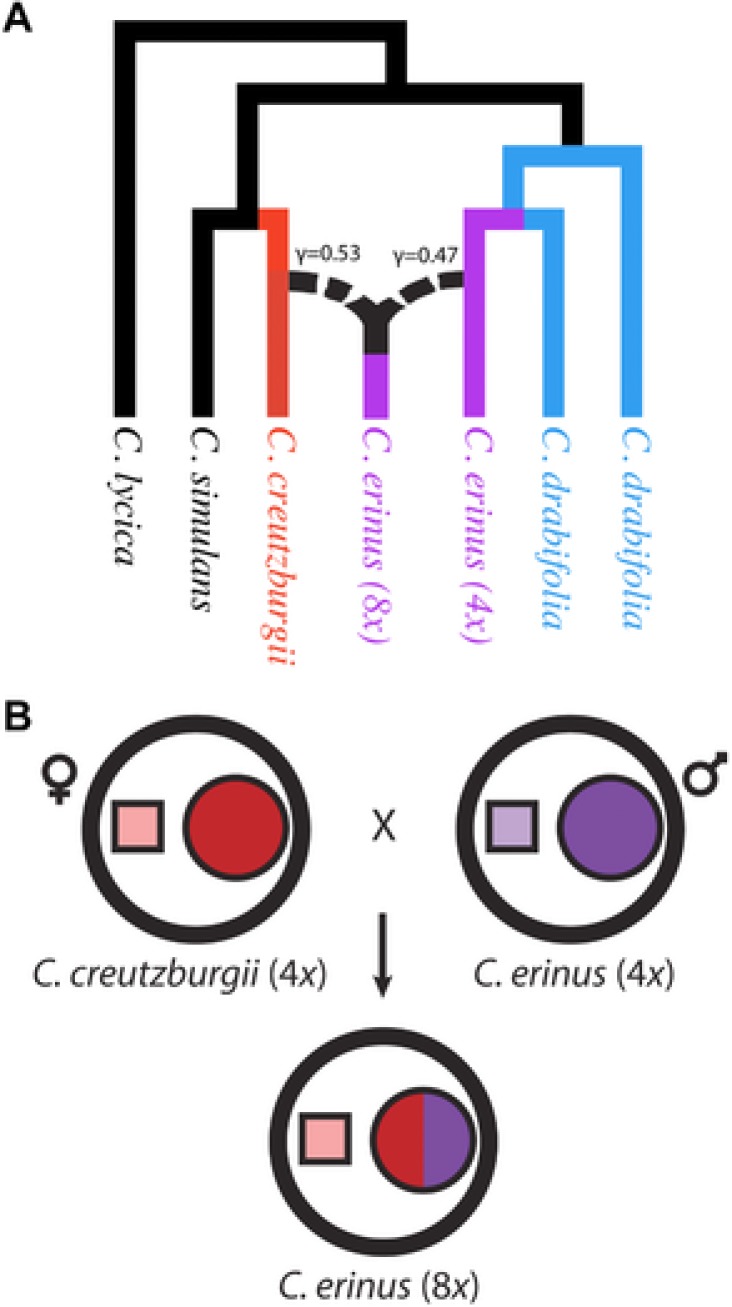
Hypothesized hybridization scenario. (A) Phylogenetic network estimated with SNaQ showing the tetraploid *C. erinus* and *C. creutzburgii* as putative parental lineages of the octoploid *C. erinus* (dashed lines). Numbers on dashed lines indicate inheritance probabilities from SNaQ analysis. These values represent the proportion of the genome estimated to have been contributed by each parental lineage. To simplify the figure, we have collapsed the *C. creutzburgii* lineages (found to be reciprocally monophyletic) to a single lineage. (B) Depiction of the hypothesized hybridization event indicating maternal and paternal genomes involved in the allopolyploid event. Large circles represent nuclear genomes while smaller squares represent plastomes. Red and purple colors indicate parental contributions from each tetraploid.

SNaQ analyses suggested a single hybridization event (Fig. 3). The best network (–loglik = 129.62) included a single hybrid edge between *C. creutzburgii* and the tetraploid *C. erinus*, indicating these as the parental lineages of the octoploid *C. erinus*. A paraphyletic *C. drabifolia* was found to be sister to the tetraploid *C. erinus*, and a monophyletic *C. creutzburgii* sister to *C. simulans*. These analyses estimated a gamma value of 0.466 for tetraploid *C. erinus* and gamma = 0.534 for *C. creutzburgii* parental lineages. Our manual inspection of gene trees yielded similar results with 49% of gene trees indicating a close association of the octoploid lineage with the tetraploid lineage and 43% indicating an association with *C. creutzburgii*. No improvement in the likelihood was observed (–loglik = 129.62) when we ran the analysis using hmax = 2 (two hybridization events).

### PLOIDY

Flow cytometry results provided easy‐to‐interpret ploidy estimates for all individuals tested. Both tetraploid (2*n* = 28) and octoploid (2*n* = 56) populations were found within *C. erinus*. The two ploidal levels correspond to geographical ranges, with all identified tetraploid populations occurring west of Greece and all octoploid populations occurring in the eastern Mediterranean Basin (Fig. 1). All *C. creutzburgii* and *C. drabifolia* populations were verified as tetraploid. Carlström (1986) cited an earlier chromosome count for *C. creutzburgii* as 2*n* = 56, but warned that this record needs confirmation as this same count had been reported for *C. erinus*, which is sympatric on the island of Crete. Our results validate this concern and we propose 2*n* = 28 may be a more accurate count for *C. creutzburgii* based on genome size estimations of five individuals from three populations, though this should be further verified.

### MORPHOLOGY

**Figure 4:**
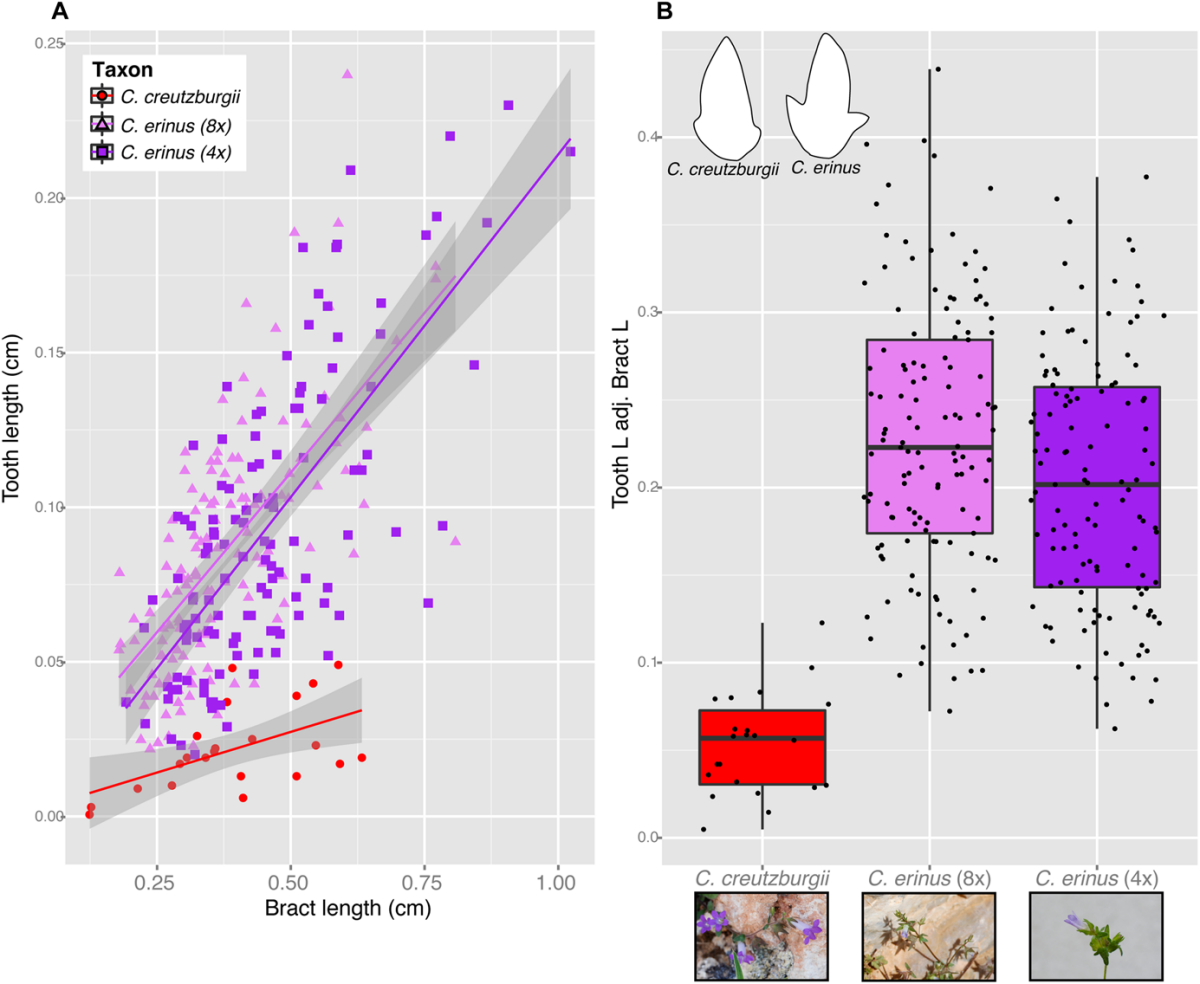
Morphology data. (A) Graph of bract tooth length versus total bract length for *C. creutzburgii* (red circles), *C. erinus* 4× (purple squares), and *C. erinus* 8× (pink triangles). Solid lines are linear regressions for each lineage, gray bars indicate 95% confidence intervals. (B) Box‐plot of bract tooth length corrected for total bract length for the three lineages. Colors consistent with those in (A). Illustrations of the different morphologies shown in upper left corner. Photos for *C. creutzburgii*, tetraploid *C. erinus*, and octoploid *C. erinus* shown below lineage name. Photos by Andrew A. Crowl.

Though molecular data and genome size estimates suggest multiple lineages within *C. erinus*, previous morphological work failed to distinguish these two groups. Our morphological dataset verifies this, indicating no discernable difference between the tetraploid and octoploid lineages based on bract tooth length (Fig. 4), suggesting these lineages represent cryptic diversity. Measurements of the bract tooth showed a clear difference between traditionally viewed *C. erinus* (both tetraploid and octoploid populations) and *C. creutzburgii* (Fig. 4). These taxa are also easily differentiated on the basis of corolla length (Carlström 1986).

## Discussion

Phylogenetic analyses, with corroboration from genome‐size estimates and morphologic data, suggest cryptic diversity is present within *Campanula erinus*, as currently recognized. Due to the highly similar morphologies of the two lineages recovered here, this diversity had, until now, been overlooked. Flow cytometry estimates of genome size found evidence for tetraploid and octoploid populations, as noted by Carlström (1986). These populations are geographically distinct, with octoploids occurring in the eastern Mediterranean—Greece, Aegean islands, and Cyprus—while the tetraploids inhabit a wide area in the western Mediterranean (Fig. 1). Unfortunately, our population sampling was insufficient to determine the precise geographic location (approximately mainland Greece or the Balkans) that demarcates the eastern distribution limit of tetraploid and the western limit of octoploid populations. More in‐depth sampling would provide this boundary and indicate whether or not the tetraploid and octoploid lineages have overlapping distributions in this region.

Conflicting phylogenetic signal between plastid and nuclear datasets, and within the nuclear dataset, appears to be the signature of hybridization. The sister relationship of the octoploid *C. erinus* lineage with the tetraploid *C. erinus* and tetraploid *C. creutzburgii*, suggests a hybrid origin for this taxon and implicates these tetraploid taxa as parental lineages. Network analyses, estimated under the coalescent while allowing for hybridization, confirm this assertion. These analyses, with corroboration from our manual inspection of gene trees, suggest the octoploid lineage of *C. erinus* was likely the result of an allopolyploid event (or events) with near‐equal contributions from the two parental lineages (Fig. 3).

The nonmonophyly of octoploid populations recovered in our phylogenetic analyses based on the concatenated nuclear dataset (Fig. 1, panel C) may be evidence of multiple polyploid origins or simply the result of incomplete lineage sorting. The results from our species‐tree analyses, unfortunately, do not satisfactorily resolve this issue. Our SVDquartets analysis in which individuals were assigned to populations recovered a maximally supported octoploid *C. erinus* clade (Fig. 2, panel D)—suggesting ILS is the cause for the nonmonophyly in the concatenation analyses, while this same assignment of individuals analyzed with ASTRAL‐II (Fig. 2, panel E) confirms the nonmonophyly of octoploid populations recovered in the concatenation analyses. A more in‐depth investigation is necessary to confirm whether a single event led to the octoploid lineage, or if polyploid formation has been recurring.

Dating analyses of Crowl et al. (2015) and Crowl et al. (2016), in which age estimates for the *Roucela* clade were inferred within the broader Campanuloideae, suggest the timing of the *C. erinus*–*C. creutzburgii* hybridization event may have coincided with the Messinian Salinity Crisis. The closure of the Mediterranean Basin's connection with the Atlantic Ocean (5.96–5.33 Ma) led to a significant desiccation of the Mediterranean Sea (Hsu et al. 1973; Krijgsman et al. 1999). This event led to the connection of previously isolated islands to each other and the mainland, potentially facilitating range‐expansion and sympatry of *C. erinus* and *C. creutzburgii*. Crowl et al. (2015) found that the relatively recent onset of the Mediterranean climate may have caused extinction in the *Roucela* complex. This provides another possible explanation for the formation of a hybrid taxon from two currently nonsympatric species, as it is conceivable that these taxa had wider distributions in the past. A more in‐depth survey of ploidal levels within populations found in Crete and mainland Greece would provide further insights into the precise mechanism underlying this apparent allopolyploid event, or events.

Though past researchers have argued that polyploidy may allow for broad ecological tolerance and, thus, broad geographic ranges (see Stebbins 1950), the narrow endemic polyploid taxa in the *Roucela* clade indicate it clearly is not polyploidy *per* se that is responsible for the wide distribution of *C. erinus*. We suggest that the distribution pattern observed within *C. erinus* is the result of two factors. First, what has been historically considered *C. erinus* is, in fact, composed of two lineages. The assertion that a single species is distributed across the Mediterranean Basin is, therefore, erroneous. Second, because the octoploid *C. erinus* appears to have retained the ability to self pollinate from the parental tetraploid *C. erinus*, this cytotype was likely able to rapidly and widely disperse across the eastern part of the basin, in contrast to the numerous narrowly distributed taxa in this same region—a result that corroborates Baker's Law (Baker 1955).

This study provides evidence for allopolyploidy resulting in cryptic diversity within a small clade of flowering plants in the Mediterranean Basin. Our results highlight the utility of target enrichment approaches for obtaining multilocus, genomic datasets for thorough assessments of species diversity and the need to carefully consider gene‐tree discordance within such datasets. Though progress has been made, much work needs to be done in regards to species assessments across the Tree of Life in this biodiversity hotspot. With the help of genomic data, future studies will surely uncover further cryptic diversity, as has been done here, providing more accurate assessments of biodiversity in this fragile region of the world.

Associate Editor: C. Ané

Handling Editor: P. Tiffin

## Supporting information


**Figure S1**. Accession information for individuals included in this study.
